# Evaluation of a novel information resource for patients with bronchiectasis: study protocol for a randomised controlled trial

**DOI:** 10.1186/s13063-016-1330-4

**Published:** 2016-04-23

**Authors:** Katy L. M. Hester, Julia Newton, Tim Rapley, Anthony De Soyza

**Affiliations:** Institute of Cellular Medicine, Newcastle University, Newcastle upon Tyne, NE2 4HH UK; Faculty of Medical Sciences, Newcastle University, Newcastle upon Tyne, NE2 4HH UK; Institute of Health and Society, Newcastle University, Newcastle upon Tyne, NE2 4AX UK; Adult Bronchiectasis service, Freeman Hospital, Newcastle Upon Tyne, NE7 7DN UK

**Keywords:** Bronchiectasis, Exacerbation, Self-management, Education, Information, Randomised controlled trial, Feasibility study, Qualitative research

## Abstract

**Background:**

There is currently little patient information on bronchiectasis, a chronic lung disease with rising prevalence. Previous work shows that patients and their families want more information, which could potentially improve their understanding and self-management. Using interviews and focus groups, we have co-developed a novel patient and carer information resource, aiming to meet their identified needs.

The aims and objectives are:

1. To assess the potential impact of the information resource

2. To evaluate and refine the intervention

3. To establish the feasibility of carrying out a multi-centre randomised controlled trial to determine its effect on understanding, self-management and health outcomes

**Methods/design:**

This is a feasibility study, with a single-centre, randomised controlled trial design, comparing use of a novel patient information resource to usual care in bronchiectasis. Additionally, patients and carers will be invited to focus groups to discuss their views on both the intervention itself and the trial process.

The study duration for each participant will be 3 months from the study entry date. A total of 70 patients will be recruited to the study, and a minimum of 30 will be randomised to each arm. Ten participants (and their carers if applicable) will be invited to attend focus groups on completion of the study visits. Participants will be adults with bronchiectasis diagnosed as per national bronchiectasis guidelines.

Once consented, participants will be randomised to the intervention or control arm using random permuted blocks to ensure treatment group numbers are evenly balanced. Randomisation will be web-based. Those randomised to the intervention will receive the information resource (website and booklet) and instructions on its use. Outcome measures (resource satisfaction, resource use and alternative information seeking, quality of life questionnaires, unscheduled healthcare visits, exacerbation frequency, bronchiectasis knowledge questionnaire and lung function tests) will be recorded at baseline, 2 weeks and 3 months.

**Discussion:**

All outcome measures will be used in assessing feasibility and acceptability of a future definitive trial. Feasibility outcomes include recruitment, retention and study scale form completion rates. Focus groups will strengthen qualitative data for resource refinement and to identify participant views on the trial process, which will also inform feasibility assessments. Questionnaires will also be used to evaluate and refine the resource.

**Trial registration:**

ISRCTN84229105

**Electronic supplementary material:**

The online version of this article (doi:10.1186/s13063-016-1330-4) contains supplementary material, which is available to authorized users.

## Background

Bronchiectasis is a chronic lung condition, characterised by dilated bronchi, leading to symptoms of breathlessness and chronic productive cough, with intermittent infective exacerbations. Bronchiectasis has various potential aetiologies including immune deficiency syndromes, chronic asthma, chronic obstructive pulmonary disease, ciliary dysfunction and post-infectious causes, yet studies have found that between a quarter and half of cases are idiopathic [1, 2]. Patients often have recurrent, costly hospital admissions, a poorer quality of life [3, 4] and clinically significant fatigue [5, 6].

Current estimates suggest a rise in prevalence in the UK and indicate a prevalence of between 43.4/100,000 in those aged 18–30 and 1239.7/100,000 in those aged 70–79 [7]. Importantly, studies demonstrate that up to 50 % of patients with chronic obstructive pulmonary disease (COPD) have co-existent bronchiectasis [8]. There are approximately 1,000,000 patients with COPD in the UK [9]; thus there is potential for a significant increase in case finding of COPD-associated bronchiectasis over the coming years.

Bronchiectasis mortality rates are approximately 50 % higher than that of uncomplicated COPD (calculated at 3 % per annum) and are increasing [10]. The prognosis varies, with a recent study of 91 patients [11] finding that the primary cause of death was usually respiratory, with survival rates of 91 % at 4 years and 68.3 % at 12.3 years. Infective exacerbations lead to significant morbidity. Previously published UK data also emphasise the burden of bronchiectasis, uncertainties in aetiology and lack of evidence for the treatments that are often used [12]. This is consistent with recently published American data on the increasing burden of bronchiectasis [13].

There is no cure for bronchiectasis. Current modalities of treatment include oral, inhaled or intravenously administered antibiotics, used both regularly and with additional courses for exacerbations. Mucolytics and regular physiotherapy are used to aid sputum clearance, and additional guidelines for investigation, diagnosis and management of bronchiectasis have been provided by the British Thoracic Society (BTS) [14]. Inappropriate antibiotic use can lead to antibiotic resistance. Conversely, not commencing antibiotics promptly can result in a severe exacerbation requiring costly hospital admission. Bronchiectasis differs from many chronic diseases in that appropriate, timely recognition of symptoms and improved management of infections could lead to increased disease stability. This could potentially lead to longer term improvement in respiratory outcomes. For example, carrying out regular chest physiotherapy and responding appropriately to symptoms of exacerbation may prevent deterioration and reduce admissions. Patient self-care therefore could make a significant difference to management.

In order to facilitate self-care, patients need to have accurate information about their condition, empowering them to recognise changes, respond to them and understand how their self-management could potentially alter their prognosis. The BTS guidelines for the management of bronchiectasis [14] recommend education of patients within their management plan. There is relatively little information produced for patients with bronchiectasis. Sources include a one-page leaflet produced by the British Lung Foundation (BLF) and limited online resources. The BTS has a brief self-management tool for bronchiectasis that is available to download. It does not serve as an information resource but is a one-page reference guide to exacerbation management.

In addition to the need for information and education being recognised by organisations such as the BTS and the BLF, a survey of 104 patients attending a specialist bronchiectasis clinic in the North East of England found 98 % felt more confident about managing their condition after receiving information and education about their treatment [15]. A study using focus groups involving patients who have bronchiectasis has highlighted lack of information as one of the perceived obstacles to self-management [16]. In addition, a pilot study of qualitative interviews with patients identified the importance of patient information in the process of developing the skills and confidence to manage and live with bronchiectasis [17, 18]. There was a strong feeling amongst participants that there was a lack of trustworthy information (from a reliable source such as their hospital, trusted specialists or organisations such as the BLF) available to them beyond that obtained in clinic. Patients felt they would benefit from a credible information source that they could continue to access outside of a specialist clinic setting. Despite this there remains a lack of development in this area to date, and many chronic conditions that are less prevalent than bronchiectasis have many more accessible resources.

Our yet unpublished qualitative study used in-depth interviews with a cohort of 26 patients and carers to identify their unmet information needs and priorities for an information resource. We have used the themes and needs identified during analysis of these interviews to develop a novel patient information resource. The content and format of the resource are based on the findings of the interviews and subsequent focus groups with patients and carers to refine the intervention.

A definitive, multi-centre trial would address the research question: Can the provision of patient-focussed information and education improve health outcomes in bronchiectasis? The rationale for the Bronchiectasis Information and Education Feasibility (BRIEF) study is that, in advance of the definitive trial, it is necessary to assess whether the proposed design for the trial is practicable and will allow the proposed outcomes to be assessed. In addition, the intervention will be evaluated and further refined for use within the definitive trial.

The trial will be conducted as an unblinded, randomised controlled trial comparing the use of the information resource as an intervention to usual care.

### Objectives

#### Primary objective

The primary objective is to conduct a feasibility study that will inform the decision of whether to proceed to the definitive randomized controlled trial (RCT) and whether any refinements to the design or conduct of that trial are warranted.

#### Secondary objective

The secondary objective is to evaluate and further refine the patient information resource and collect information on patient preferences.

#### Definitive trial objectives

The definitive trial objectives are to assess whether provision of a patient-focussed information and education resource can improve patient understanding, self-management and health outcomes in bronchiectasis.

## Methods/design

### Participants, interventions and outcomes

#### Study setting

This is a single-centre study taking place in the UK in the Newcastle upon Tyne Hospitals NHS Foundation Trust. This consists of two teaching hospital sites: the Freeman Hospital and the Royal Victoria Infirmary. Study visits will all take place within the Freeman Hospital. The running of the trial will be based within the Freeman Hospital at the William Leech Clinical Trials Centre. Patients will be recruited from either hospital site.

#### Eligibility criteria

##### Inclusion criteria

To fulfil the inclusion criteria, the participant must:

1. Have the capacity to provide written informed consent
2. Be aged 18 years or older
3. Have received a clinical and radiological diagnosis of bronchiectasis
4. Be English speaking

##### Exclusion criteria

The exclusion criteria include:

1. Cognitive impairment
2. Non-English speaking
3. Age <18 years
4. Participation in the preceding Bronchiectasis Information and Education (BRIE) study

Due to the nature of the study, knowledge of the English language is a necessary inclusion criterion to ensure usability of the information provided. As this is a small feasibility study, resources are not currently available to produce the information in other languages or to provide funded Internet access. For those potential participants who do not have Internet access yet wish to take part in the study, the information within the website (excluding video clips) can be provided in PDF format. This will be recorded on the Case Report Form (CRF), and the participant will proceed with the same visits and outcomes.

#### Study intervention details

The BRIEF study will compare the patient information resource (developed within the previously conducted qualitative study) with usual care. At the baseline visit, participants randomised to the intervention will receive the patient information resource: an overview booklet and website. Verbal and written instructions will be given by appropriate members of the research team (as per the delegation log) about how to access the website. The participants will then have access to the intervention for the duration of the study. Their use or not of the information resource will be down to individual choice, yet through discussion with the research team member conducting each study visit participants will be encouraged to utilise the resource and to allow their families or carers to utilise it also should they so wish. Some participants may not have direct access to a computer or Internet use. This does not preclude them from entry as long as they can access the Internet via their family, friends or local institutions such as libraries. For those who do not wish or do not have the skills to access the website, participation using a PDF version of the information contained within the website will be offered. This will enable them to view all information except the video clips. At study completion, those randomised to the intervention group will be allowed continued access to the resource. Those in the control group will be offered access to the resource following completion of their study period so as to minimise disappointment due to their allocation to the control arm. Any uptake of the resource following study completion does not form part of data collection.

This is a non-clinical intervention, and we do not expect any reasons for discontinuing the intervention other than participant preference. Any participant may choose to leave the study at any point with no effect on usual care. Potential participants currently participating in another trial would not be approached for entry into the study.

#### Study design and outcome measures

**Fig. 1:**
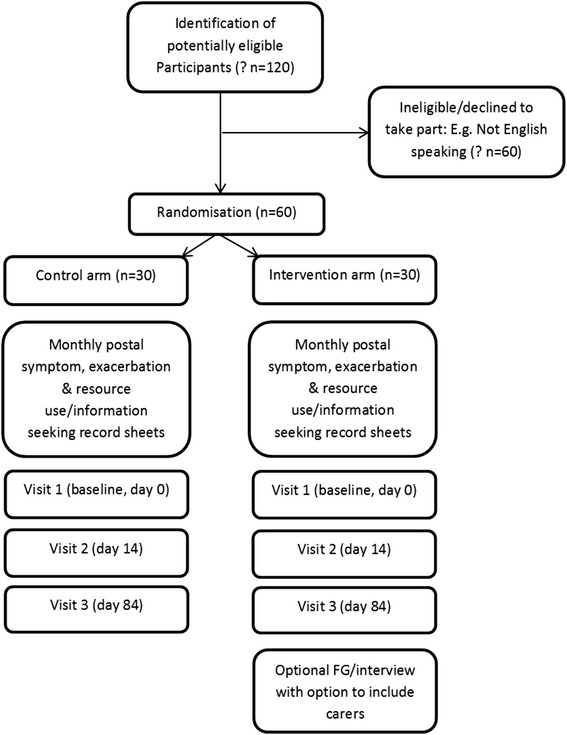
Study flow chart

See Fig. 1 and Table 1.

**Table 1:** Study visits and data collection

	Visit 1 Day 0	Visit 2 Week 2 (Day 14)	Visit 3: final visit Week 12 (Day 84)
Written informed consent and randomisation (if not done prior to visit 1) and collection of baseline demographics	x		
Resource use (not baseline visit) and information seeking	(x)	x	x
Resource satisfaction questionnaire		x	x
Bronchiectasis knowledge questionnaire	x	x	x
QOL-B	x	x	x
SGRQ	x		x
HADS	x	x	x
FIS	x		x
EQ-5D	x	x	x
Number of unscheduled visits	x	x	x
Exacerbation frequency	x	x	x
FEV1	x (if not done in past 3 months)		x

*QOL-B* Quality of Life Questionnaire-Bronchiectasis, *SGRQ* St. George’s Respiratory Questionnaire, *HADS* Hospital Anxiety and Depression Scale, *FIS* Fatigue Impact Scale, *EQ-5D* Euroqol 5-dimension (quality of life scale), *FEV1* forced expiratory volume in 1 second

##### Design

This feasibility study is an unblinded, single-centre randomised controlled trial with two parallel groups that compares a novel patient information resource to usual care in bronchiectasis.

##### Duration

The study duration for each participant will be 3 months from the study entry date. Due to variations in month length, this will be calculated at 12 weeks (84 days). The study completion date will be after the date of the last assessment visit of the last entrant and completion of the final focus group.

##### Outcome measures

The outcome measures are as follows:

1. Ability to recruit adequate numbers of participants (ratio of consented participants to potentially eligible participants approached)
2. Numbers completing study
3. Numbers completing study scale forms, questionnaires and visits
4. Resource satisfaction questionnaire
5. Recorded use of resource and alternative information seeking
6. Quality of Life Questionnaire-Bronchiectasis (QOL-B)
7. St George’s Respiratory Questionnaire (SGRQ)
8. Hospital Anxiety and Depression Scale (HADS)
9. Fatigue Impact Scale (FIS)
10. Euroqol 5-dimension (quality of life scale), EQ-5D
11. Number of unscheduled visits to primary or secondary care
12. Exacerbation frequency
13. Forced expiratory volume in 1 second (FEV1)
14. Knowledge of condition and management questionnaire

All outcome measures will be used in assessing the feasibility of a future definitive trial, including recruitment, retention and study scale form completion rates. Participant evaluation of acceptability of the newly developed package will be established through a questionnaire and open questioning to include their satisfaction with the information provided, knowledge about their condition and management, and additional features that they feel may strengthen the intervention. Use of the resources provided and preferred formats identified within these questionnaires will also inform feasibility of a future trial and allow refinement of intervention formats. Focus groups will also be held to strengthen data on the patient experience.

Outcome measures will be recorded at baseline (day 0) and then at 2 weeks (day 14) (that is, shortly after initial viewing of information in order to facilitate obtaining first opinions) and 3 months (day 84) post recruitment. This will be done during patient visits that are anticipated to take less than 1 hour each. Visit 2 can be done via telephone interview if participants prefer.

The time taken to complete the scales and estimates of variability in outcome measures for the population at the various time points, with associated confidence intervals, will help to inform a future sample size calculation for a definitive RCT. We will describe these data as a reference for this patient group and as baseline measures to inform a future RCT. We will examine the relationship between outcomes and baseline covariates in order to identify any efficacy gains through the use of stratification in a future full RCT.

EQ-5D will be used to allow some estimate of health economic evaluation for a future RCT. We anticipate potential health economic benefits with patients empowered to self-manage, thereby reducing service use. The number of unscheduled presentations, exacerbation rate and FEV1 could potentially be used in a future full trial as a representation of the patients’ ability to self-manage their condition. This information will be retrieved from the patient visits and patients’ symptom and information sheets (patients will be asked to complete a monthly postal record sheet [at weeks 4, 8 and 12] enabling identification of episodes of change in symptoms and actions taken, in addition to any information resource use, without the burden of a daily diary record) and also through general practitioner (GP) and hospital recorded attendances if patients are unable to report or recollect.

#### Participants

Potential participants will be identified by case note review and attendance at outpatient clinics and will be given or sent a letter of invitation to the study and a patient information sheet. Written informed consent will be obtained from willing participants (see Additional file 1). Patients can withdraw consent at any point with no effect on usual care. At the end of the study, some participants will be invited to attend a focus group and a possible in-depth interview about their experience. For this section of the study only, if participants in the intervention group indicate that their partner/family member or friend has also used the resource, then they may be invited to attend discussion groups also. Up to a maximum of 10 additional participants will be recruited for this purpose. Additional information sheets and consent forms have been produced for these participants (see Additional file 2). Participants invited to attend the focus group will be sampled purposively. The intent is to form a group that includes participants of differing backgrounds and time since diagnosis, some from the control and some from the intervention group, and those who had differing preferences in terms of format used. Involvement in the focus group, however, is an optional extra, and as such a pragmatic approach will have to be taken. Anyone agreeing to take part in the focus group will be invited to bring along their ‘carer’, who will then be sent the appropriate information sheet to consider whether they would like to take part.

#### Participant identification and screening

Patients will be identified through case note review and clinic attendance. Eligible participants will be invited to participate by their consultant, the principal investigator (PI) or the chief investigator (CI), who is part of the medical team. The study will be explained to them further by the research team. A study Participant Information Sheet will be provided at this time, which the patient can take away for consideration. For those identified by case note review, a letter of invitation will be sent in the post along with the Participant Information Sheet and details of how to get in touch if interested. They will be offered the opportunity to discuss this further with the research team.

A screening log will be kept to document details of subjects invited to participate in the study. For subjects who decline participation, this will document any reasons available for non-participation. The log will also ensure potential participants are only approached once.

#### Sample size

The sample size will be 70, with a minimum of 30 being randomised to each arm. This is based on previous recommendations for good practice in feasibility studies [19]. Because this is a feasibility study, no formal power calculations have been carried out. Up to 10 non-patient (carer) participants will be recruited for the qualitative section of the study, as discussed in the section ’Focus group data’.

We anticipate that 24 months will be adequate time to recruit 70 patients to this study, based on a clinic attendance of approximately 60 per month with an estimate of 50 % of patients approached who are willing and able to enter. Seventy patients recruited from approximately 140 patients approached would correspond to a 95 % confidence interval for the recruitment rate of 41–59 % (an acceptable width of ±9 %). We expect low attrition rates based on previous work and our prior experience in this field. There will be a 3-month additional period for follow-up of the last recruited participants and time beyond for the interviews, focus groups and analysis.

### Assignment of intervention

#### Randomisation

Participants will be randomised to intervention or control in a 1:1 ratio, using random permuted blocks within strata. Randomisation will be stratified by gender. The randomisation allocation schedule will be generated by a statistician with no other involvement in the study. Randomisation will be performed by the CI at site, or an individual with delegated authority, using a secure password-protected web-based system administered by Newcastle Clinical Trials Unit. Randomisation will generate a unique 3-digit study identification number for each participant. Participants will be informed of their allocated treatment group following randomisation. Blinding is not feasible for this study for patients or the research team conducting the study visits due to the nature of the intervention. The data analyst is also involved directly with the study processes and data collection, and thus blinding of the analyst is not possible.

### Data collection, management and analysis

#### Data collection

Data will be collected at study visits by the research team as per the delegation log. Visits 1 and 3 will be done in person; visit 2 can be either in person or on the telephone. Other than breathing tests at visits 1 and 3, all outcome measures are questionnaires and will be either self-completed by the participants or completed with the help of the research team member conducting the study visit. All answers will be recorded in paper copies of each questionnaire within the CRF. The study team member conducting the visit will check for omissions after completion with the participant. All members of the delegate log will be trained in the use of the questionnaires and lung function tests. The questionnaires to be completed are summarised in Table 2. Additional data collection will be obtained via monthly (weeks 4, 8, 12) postal symptom and resource use record sheets sent to participants. This will enable more accurate recollection of symptoms and information use than at the study visits alone, yet is a reduction in burden as compared to completing a daily diary. Phone calls will be made to encourage completion if the forms are not returned.

**Table 2:** Outcome measures

Study instrument	Description
Resource use and information seeking	Unvalidated questionnaire
Resource satisfaction questionnaire	Unvalidated questionnaire
Bronchiectasis knowledge questionnaire	Unvalidated questionnaire
QOL-B	Validated Quality of Life Questionnaire-Bronchiectasis [26]
SGRQ	Validated St George’s Respiratory Questionnaire [3]
HADS	Validated Hospital Anxiety and Depression Scale [27]
FIS	Validated Fatigue Impact Scale [28]
EQ-5D	Validated Euroqol 5-dimension quality of life questionnaire [29]
Number of unscheduled visits	Patient’s report of healthcare visits
Exacerbation frequency	Patient’s report of number of exacerbations
FEV1 (absolute value and % predicted)	Lung function test (forced expiratory volume) using calibrated equipment

#### Focus group data

A number of participants will be invited to a focus group to discuss participation in the trial and views on further refinements to the intervention and the study protocol. Should specific issues arise within the focus groups that need further exploration, then a number of in-depth interviews may also be held. These would be entirely optional. If participants indicate that their partner, family member or friend has used the resource, then they may be invited to this section of the study also. A maximum of 10 carers will be recruited and given the appropriate Participant Information Sheet, and written informed consent will be obtained. Thematic analysis will be used to look for patterns of meaning and ‘themes’ in the data content. Data will be organised into meaningful groups to identify and describe themes and issues raised in the interviews.

#### Data handling and record keeping

Data collected on paper CRFs will be entered by the CI or appropriately trained study delivery staff and data manager (as per the delegation log) on a secure password-protected study computer. Participants will be identifiable only by a unique study identifier on all recorded data.

Focus group audio files will be transcribed verbatim. All data will be stripped of strong identifiers and will only be identified by a unique study number, and only authorised members of the research team, operating to written codes of confidentiality, will have access to the link between anonymised data and patient/professional identifiable details. Patients and professionals will not be identifiable in any publications emanating from the work described in this application. Data will be handled, computerised and stored in accordance with the Data Protection Act 1998. No participant identifiable data will leave the study site.

#### Compliance and withdrawal

##### Compliance

Where feasible, study visits will coincide with routine clinical follow-up to enhance the likelihood of good compliance. Visit windows of 2 weeks should ensure visit attendance; non-attendance for study visits will prompt follow-up by telephone. Participants will be given the option of completing visit 2 by telephone interview to reduce the burden of travel for study visits. Non-return of monthly postal record sheets will also prompt follow-up by telephone.

##### Withdrawal of participants

Participants have the right to withdraw from the study at any time for any reason and without giving a reason. The investigator also has the right to withdraw patients from the study intervention if she/he judges this to be in the patient’s best interests. It is understood by all concerned that an excessive rate of withdrawals can render the study uninterpretable; therefore, unnecessary withdrawal of patients should be avoided. Should a patient decide to withdraw from the study, all efforts will be made to report the reason for withdrawal as thoroughly as possible.

There are two withdrawal options:

1. Withdrawing completely (withdrawal from both the study intervention and provision of follow-up data)
2. Withdrawing partially (withdrawal from the study intervention but continuing to provide follow-up data by attending clinic and completing questionnaires)

Consent will be sought from participants choosing option 1 to retain data collected up to the point of withdrawal. Participants will be asked if they would allow the reason for the decision to withdraw to be recorded.

#### Statistical analysis

A statistical analysis will be performed using SPSS 17.0. As this is a feasibility study, the analyses of the data collected will be mainly descriptive, with 95 % confidence intervals reported where appropriate. As a randomised controlled trial, the primary analysis will be based on the intention-to-treat principle with analysis groups based on the groups allocated at randomisation and all randomised patients being included in the analysis. As a feasibility study, the extent of missing data will be assessed and reported, and analysis of outcomes may also be carried out on a complete-case basis. Rates will be calculated as defined and reported with 95 % confidence intervals. At baseline and by intervention group the distribution of all numerical variables will be examined and summarised using measures of location and spread. Similarly, baseline categorical variables will be tabulated and percentages reported. Change in the questionnaire outcomes from baseline to 2 weeks and 3 months will be summarised. The difference in the mean change between the intervention groups from baseline to each of the two time points will be explored for all outcome measures and reported with accompanying 95 % confidence intervals. Such results will be interpreted cautiously because of the size of the study and the possible imbalance in pre-randomisation baseline covariates. The relationship between baseline covariates and outcome measures will be examined graphically and quantified appropriately depending on their distribution. No formal statistical testing will be performed. Confidence limits for the estimated standard deviations of key study parameters will be calculated and used in sensitivity analyses for sample size calculations for a future definitive RCT.

### Monitoring

This is a low risk trial, and major safety data are not anticipated. As agreed upon by Newcastle upon Tyne Hospitals, the Trial Oversight Committee (TOC) will adopt the joint roles of Trial Steering Committee (TSC) and Data Monitoring and Ethics Committee (DMEC) with independent members meeting in closed session to fulfil the DMEC role. The TOC comprises an independent chair, an independent consumer representative, a patient representative, a carer representative, CI, PI, data manager and statistician. The TOC will meet bi-annually. Their role is to monitor progress and supervise the trial to ensure it is conducted to high standards in accordance with the protocol, the principles of good clinical practice (GCP) and relevant regulations and guidelines and with regard to participant safety. The purpose of this committee will be to monitor study progress and patient safety. Monitoring of study conduct and data collected will be performed by site review to ensure the study is conducted in accordance with GCP. The main areas of focus will include consent, serious adverse events and essential documents in study.

The study may be subject to inspection and audit by Newcastle upon Tyne Hospitals under their remit as sponsor and by other regulatory bodies to ensure adherence to GCP. The investigator(s)/institutions will permit trial-related monitoring, audits, Research Ethics Committee (REC) review and regulatory inspection(s), providing direct access to source data/documents.

There is no interim analysis planned for this study.

### Adverse event monitoring and reporting

#### Definitions

##### Adverse event (AE)

An AE is an untoward medical occurrence in a subject to whom a study intervention or procedure has been administered, including occurrences which are not necessarily caused by or related to that intervention. An AE, therefore, does not necessarily have a causal relationship with the treatment. In this context, ‘treatment’ includes all interventions (including comparative agents) administered during the course of the study. Medical conditions/diseases present before starting study treatment are only considered AEs if they worsen after starting study treatment.

##### Related adverse event

A related AE is one that results from administration of any of the research study procedures. All AEs judged by either the reporting investigator or the sponsor as having reasonable causal relationship to a study procedure qualify as ‘related adverse events’. The expression ‘reasonable causal relationship’ means to convey in general that there is evidence or argument to suggest a causal relationship.

##### Causality

The assignment of the causality should be made by the investigator responsible for the care of the participant using the definitions in Table 3. All AEs judged as having a reasonable suspected causal relationship to a study procedure (that is, definitely, probably or possibly related) are considered to be related AEs. If any doubt about the causality exists, the local investigator (PI) should inform the CI. In the case of discrepant views on causality between the investigator and others, all parties will discuss the case. In the event that no agreement is made, the main REC and other bodies will be informed of both points of view.

**Table 3:** Definitions of causality

Relationship	Description
Unrelated	There is no evidence of any causal relationship
Unlikely	There is little evidence to suggest there is a causal relationship (e.g. the event did not occur within a reasonable time after administration of the study procedure). There is another reasonable explanation for the event (e.g. the participant’s clinical condition or other concomitant treatment)
Possible	There is some evidence to suggest a causal relationship (e.g. because the event occurs within a reasonable time after administration of the study procedure). However, the influence of other factors may have contributed to the event (e.g. the participant’s clinical condition or other concomitant treatments)
Probable	There is evidence to suggest a causal relationship and the influence of other factors is unlikely
Definitely	There is clear evidence to suggest a causal relationship and other possible contributing factors can be ruled out
Not assessable	There is insufficient or incomplete evidence to make a clinical judgement of the causal relationship

### Unexpected adverse event

An unexpected AE is one that is not listed in the study protocol as an expected occurrence in the circumstances of this trial.

#### Serious adverse event (SAE)

An SAE is an untoward occurrence (whether expected or not) that:

- Results in death
- Is life-threatening (refers to an event in which the subject was at risk of death at the time of the event; it does not refer to an event which hypothetically might have caused death if it were more severe)
- Requires hospitalisation or prolongation of existing hospitalisation
- Results in persistent or significant disability or incapacity
- Consists of a congenital anomaly or birth defect
- Is otherwise considered medically significant by the investigator

Medical judgement should be exercised in deciding whether an AE is serious in other situations. Important medical events that are not immediately life-threatening or do not result in death or hospitalisation but may jeopardise the patient or may require intervention to prevent one of the other outcomes listed in the definition above should also be considered serious.

### Severity (intensity) of adverse events and adverse reactions

The severity of all AEs will be graded on a three-point scale of intensity (mild, moderate and severe):

- Mild: Discomfort is noticed, but there is no disruption of normal daily activities.
- Moderate: Discomfort is sufficient to reduce or affect normal daily activities.
- Severe: Discomfort is incapacitating, resulting in inability to work or to perform normal daily activities.

An AE may be severe but not serious.

### Expected adverse reactions

This is a low risk study, and there are no expected adverse reactions (ARs) from the intervention as it is an information resource rather than a treatment. Study procedures in the main are completing forms. Very occasionally when people perform spirometry (which will be measured at study visits) they may feel light headed for a short while afterwards. Spirometry tests will be performed seated, and if participants have a known tendency, this test will be omitted. As this is a rare but expected AR, it would not be reported. Only suspected unexpected serious adverse reactions (SUSARs) will be reported.

### Recording and reporting adverse events or reactions

All AEs should be reported as per protocol specifications. Depending on the nature of the event, the reporting procedures in the succeeding paragraphs should be followed. Any questions concerning AE reporting should be directed to the CI or PI in the first instance.

#### Adverse events

All non-serious AEs during study participation will be reported on the study CRF and sent to the CI within 2 weeks of the form being due. Severity of AEs will be graded on a three-point scale (mild, moderate and severe). Relation (causality) and seriousness of the AE to the treatment should be assessed by the investigator at site in the first instance. The individual investigator will be responsible for managing all AEs according to local policy.

#### Serious adverse events

All SAEs during study participation shall be reported to the CI within 24 hours of the site learning of its occurrence. The initial report can be made by telephone or email. Use of the SOHO66 fax system ensures that the NCTU, sponsor and CI are all informed by email simultaneously.

In the case of incomplete information at the time of initial reporting, all appropriate information should be provided as follow-up as soon as it becomes available. The relationship of the SAE to study procedures should be assessed by the investigator at site, as should the expected or unexpected nature of the SAE.

### Ethics and dissemination

#### Ethics and regulatory issues

The conduct of this study will be in accordance with the recommendations for physicians involved in research on human subjects adopted by the 18th World Medical Assembly, Helsinki, 1964 and later revisions.

Favourable ethical opinion from NRES Committee North East - Sunderland (reference 14/NE/0119) has been granted, and R&D approval (reference 7005) from the Newcastle upon Tyne Hospitals NHS Foundation Trust was granted prior to commencement of the study. Any protocol amendments will be approved by R&D and the Sunderland REC and will be communicated to all relevant parties: investigators, registries, participants.

Information sheets will be provided to all eligible subjects and written informed consent obtained prior to any study procedures.

### Informed consent procedures

Informed consent discussions will be undertaken by appropriately trained site staff (as per the delegation log) involved in the study, including medical staff and research nurses, with the opportunity for participants to ask any questions. Following receipt of information about the study, participants will be given reasonable time (aiming for a minimum of 24 hours) to decide whether or not they would like to participate. Those wishing to take part will provide written informed consent by signing and dating the study consent form, which will be witnessed and dated by a member of the research team with documented, delegated responsibility to do so. Written informed consent will always be obtained prior to randomisation and prior to study-specific procedures/investigations.

The original signed consent form will be retained in the Investigator Site File, with a copy in the clinical notes and a copy provided to the participant. The participant will specifically consent to his/her GP being informed of their participation in the study.

The right to refuse to participate without giving reasons will be respected.

Due to the small subject population and the inclusion criteria, the information sheet and consent form for the study will be available only in English.

### Confidentiality

Personal data will be regarded as strictly confidential. To preserve anonymity, any data leaving the site will identify participants by their initials and a unique study identification code only. The study will comply with the Data Protection Act 1998. All study records and Investigator Site Files will be kept at site in a locked filing cabinet with restricted access. Only members of the research team will have access to the final dataset and access as required for necessary audit and monitoring.

### Insurance and finance

The sponsor, the Newcastle upon Tyne Hospitals NHS Foundation Trust, has liability for clinical negligence that harms individuals toward whom they have a duty of care. NHS indemnity covers NHS staff and medical academic staff with honorary contracts conducting the trial for potential liability in respect of negligent harm arising from the conduct of the study. The Newcastle upon Tyne Hospitals NHS Trust is sponsor and, through the sponsor, NHS indemnity is provided in respect of potential liability and negligent harm arising from study management. Indemnity in respect of potential liability arising from negligent harm related to study design is provided by NHS schemes for those protocol authors who have their substantive contracts of employment with the NHS and by Newcastle University insurance schemes for those protocol authors who have their substantive contract of employment with the university. This is a non-commercial study, and there are no arrangements for non-negligent compensation. Newcastle University provides insurance coverage for the trial design.

The National Institute for Health Research (NIHR) is funding the study through a doctoral research fellowship awarded to the CI.

### Study reporting and publications

It is planned to publish this study in peer-reviewed articles and to present data at national and international meetings. Results of the study will also be reported to the sponsor (Newcastle upon Tyne Hospitals) and funder (NIHR) and will be available on their websites. All manuscripts, abstracts or other modes of presentation will be reviewed by the Trial Overview Committee and funder prior to submission. This will also form part of the PhD thesis of the CI. Individuals will not be identified from any study report. Participants will be informed about their contribution to the study, including a lay summary of the results, at the end of the study.

## Discussion

This study is a low risk study. As the study and intervention have been co-developed with potential users, we hope this will make the trial process and use of the resource straightforward and beneficial. The study will both inform a future multi-centre trial and allow for evaluation and refinement of the patient information resource to maximise potential future uptake and impact. Analysis of outcome measures will begin to determine impact of this novel information resource on patient knowledge and confidence to self-manage and inform development of a definitive trial to determine the effect on disease stability.

The relatively short duration of the feasibility study may limit the usefulness of exacerbation frequency data in terms of defining this rate for a definitive trial. We are collecting these data to determine the feasibility of their use as an outcome measure, yet for a definitive trial with a longer follow-up period, it is likely that we would need to refer to previously published rates of exacerbation frequency in bronchiectasis. Within a recent analysis of 155 patients at our centre this was found to be roughly 4 per annum [20], although this does differ between publications as outlined in the BTS guidelines for the treatment of bronchiectasis [14]. It has also been demonstrated that exacerbation rates vary seasonally [21], and with a shorter feasibility study frequency is therefore harder to extrapolate accurately. Additionally, in terms of reporting of exacerbations, within this study design we have relied upon patient reporting of numbers of exacerbations for which they have required treatment with a course of antibiotics. We have not included a strict definition of an exacerbation within the protocol as we have not asked patients to report changes in symptoms in real time nor made decisions about diagnosis of exacerbations or treatment for them. We do not anticipate a significant problem with ascertainment bias, yet consideration of this will also help to inform the definitive trial. Due to the short amount of time between visits in this study, we do not anticipate issues with recall, although patient notes can be used as an alternative mechanism.

One of the potential impacts of the intervention will be to improve symptom recognition and management. This will not, however, be a uniform change. Some participants were likely to have under-recognised exacerbations prior to study participation, and others were possibly taking courses of antibiotics for symptoms they felt were indicative of an exacerbation that were in fact just ‘normal’ variations in their chronic symptoms. Thus, using number of courses of antibiotics or change in number is not necessarily a useful measure; hence the additional recording of unscheduled healthcare usage.

The addition of qualitative data through the use of focus groups will allow for a richer exploration of the experience of both the trial process and use of the information package for participants. Although we know that patients want more information about bronchiectasis [17], it is argued that health information alone does not necessarily produce changes in behaviour [22]. However, asthma studies have shown that delivering education about the key aspects of the condition, allowing patients to acquire skills, and education in combination with clinical review and action plans can lead to demonstrable improvements [23–25]. By providing improved educational interventions that meet the needs of patients with bronchiectasis and their carers, we aim to facilitate improvements in self-management. Our aim is that in addition to providing a resource that is a reassuring source of support for patients and carers, improvements in understanding and management will lead to improvements in health outcomes and healthcare service use longer term. By evaluating and refining this patient-driven intervention and assessing the feasibility of conducting a future definitive RCT, we are making important progress in the provision of much needed interventions in bronchiectasis.

### Trial status

The study is still recruiting at time of submission. The first recruit was on 10/6/2014.

## Additional files

Additional file 1: Patient consent form. (DOC 103 kb) Additional file 2: Carer consent form. (DOC 102 kb)

## Abbreviations

AE: adverse event
AR: adverse reaction
CI: Chief Investigator
CRF: Case Report Form
DMEC: Data Monitoring and Ethics Committee
EQ-5D: Euroqol 5-dimension (quality of life scale)
FEV1: forced expiratory volume in 1 second
FIS: Fatigue Impact Scale
GCP: Good Clinical Practice
HADS: Hospital Anxiety and Depression Scale
MREC: Main Research Ethics Committee
NCTU: Newcastle Clinical Trials Unit
NIHR: National Institute for Health Research
NUTH: Newcastle upon Tyne Hospitals
PI: Principal Investigator (at each site)
QOL-B: Quality of Life Questionnaire-Bronchiectasis
R&D: research and development
RCT: randomised controlled trial
SAE: serious adverse event
SGRQ: St George’s Respiratory Questionnaire
SUSAR: suspected unexpected serious adverse reaction
TOC: Trial Oversight Committee
UP: unscheduled presentation to primary or secondary care
