# Supplementary material for: Evaluation of a novel information resource for patients with bronchiectasis: study protocol for a randomised controlled trial
Source: Trials. 2016 Apr 23;17:210. doi: 10.1186/s13063-016-1330-4 (PMC4841977; doi:10.1186/s13063-016-1330-4)
Supplement: Additional file 2: — Carer consent form. (DOC 102 kb) [file 13063_2016_1330_MOESM2_ESM.doc]

**DEPARTMENT OF RESPIRATORY MEDICINE**


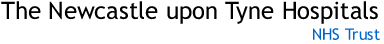

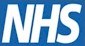


Freeman Hospital

High Heaton

Newcastle upon Tyne

NE7 7DN

Sir William Leech Centre for Lung Research


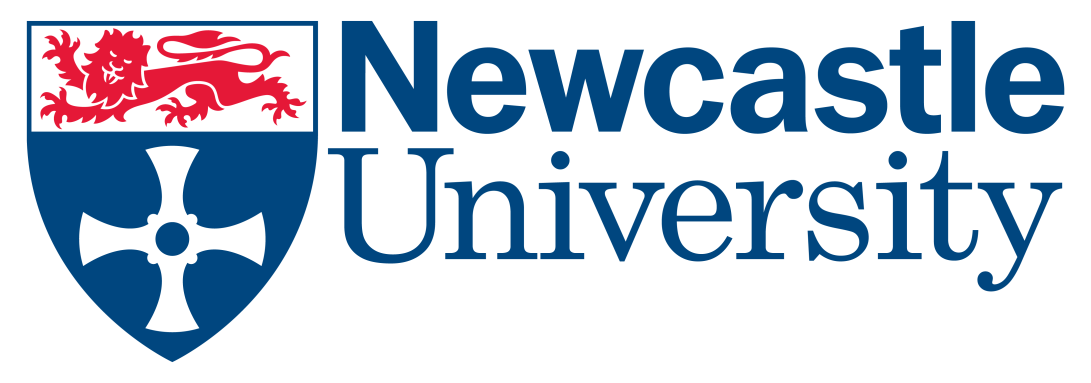


# **PARTICIPANT INFORMED CONSENT FORM**

# **(Focus group only)**

**Short Title: Bronchiectasis information and Education Feasibility (BRIEF)**

**Researchers: Dr Katy Hester, Dr Tim Rapley, Prof Julia Newton, Dr Anthony De Soyza**

**Please initial boxes**

**Participant initials: Participant code number:**

**1.** I confirm that I have read and understand the information provided in the participant information sheet, dated **23^rd^ January 2014, version 1.0**. The information has been explained to me, I have been given the opportunity to ask questions and I am satisfied with the explanations provided.

**2**. I am aware that my participation in this study is entirely voluntary. I understand that I may withdraw at any time, without giving a reason, without this affecting my future care or legal rights.

**3**. I understand that relevant sections of any of my medical notes and data collected during the study may be looked at by responsible individuals from the research team, The Newcastle upon Tyne NHS Hospitals Foundation Trust, or from regulatory authorities where it is relevant to my taking part in this research. I give permission for these individuals to have access to my records.

**4**. I understand that an audio recording will be made of my interview that will only be used for the purposes of this research and agree to this being done.

**6**. I understand that I will receive a signed copy of this consent form.

**7**. I agree to take part in this study.

**Signatures**

**Name of participant: Date: Signature:**

**Name of person taking consent:** **Date:**  **Signature:**
